# Filling Teeth When the Lining Membrane Is Exposed

**Published:** 1851-10

**Authors:** Chapin A. Harris


### ARTICLE VII.

*Filling Teeth when the Lining Membrane is Exposed.* By CHAPIN A. HARRIS, M. D.

IN the July number of the Journal for 1849, I took occasion to offer a few remarks on filling teeth after the lining membrane had become exposed, giving at the same time, a brief history of the operation, together with the result, in very general terms, of my experience and observations upon the subject. About a year after, I treated of it in another place, at somewhat greater length,* but without entering much into detail. Within the last few months it has attracted the attention of several members of the profession, and the practicability of performing it successfully, does not now seem to be regarded with as much distrust as it was a few years ago. But, before entering upon the discussion of the subject, it may be well to recapitulate, in a few words, what was said in my former article, concerning the history of the operation.

In his "Principles of Dental Surgery," published in 1822, Dr. L. Koecker recommends filling teeth after the lining membrane has become exposed, saying, he had practiced the operation successfully for thirteen years.

The method of procedure pursued by Dr. K., consists, first, in the removal of the decayed part of the tooth, then, after wiping out the cavity with raw cotton, moistened in warm water, covering the exposed pulp with a piece of sheet lead, and introducing in the usual way, a filling of gold. His reason for placing sheet lead in the bottom of the cavity previous to the introduction of the gold, was this : he believed that the former metal had a more cooling and anti-inflammatory effect than the latter. When he wounded the lining membrane in removing the diseased bone, he cauterized the part with a red hot iron wire. He speaks of the practicability of performing the operation successfully, in the most confident terms, expressing the

* See 4th Edition of Principles and Practice of Dental Surgery.

belief that five teeth out of every six, may be preserved alive by it, and rendered useful for many years. He also affirms that he had succeeded in preserving the vitality of teeth after they had ached, and the lining membrane had become the seat of inflammation.

It is scarcely necessary to observe, that in the hands of other practitioners, the method of procedure recommended by Dr. Koecker, has not proved so successful. It has been, therefore, I believe, wholly abandoned.

Dr. Fitch recommends in his "Dental Surgery," placing a cap of gold over the pulp, previously to filling the tooth, and this has been found to succeed much better than lead.* But where the cavity is very shallow, it occupies so much space as to render the introduction of a good filling, exceedingly difficult, and sometimes impossible. Nor can a gold cap, in the majority of cases, be fitted with sufficient accuracy to the various irregularities of the walls of the cavity, to prevent it from being moved or partially displaced by the subsequent introduction of a filling. Besides, I am not convinced that any advantage can be derived from it, under any circumstances whatever. On the contrary, I believe a tooth can be filled over an exposed pulp, with greater certainty of success without it, than with it. At any rate, my own experience justifies the conclusion. I have performed the operation during the last sixteen years, in some hundreds of cases, and not in a single one, have I deemed it necessary to cover the pulp with a cap previously to the introduction of the filling.

Although the practicability of filling a tooth after caries has penetrated to the pulp-cavity, without causing pain, and *immediate* subsequent inflammation and suppuration of the lining membrane and pulp, is now so well established as scarcely to admit of doubt, the possibility of thus preserving their vitality for any considerable length of time, is still regarded by many

* Since the publication of my former article on filling teeth, when the lining membrane is exposed, I have been informed that a gold cap for protecting the pulp, had been used several times by Dr. L. S. Parmly, before the publication of Dr. Fitch's work.

respectable practitioners, as somewhat questionable. And, notwithstanding the apparent success of the operation in my own practice, I confess, I was unable to explain satisfactorily to my own mind, how a vacant space could exist between the filling and lining membrane, without causing some prejudicial effect upon the latter. That some hidden or mysterious resource of the economy was called into operation for its prevention, seemed very probable, but, until recently I could not exactly understand in what it consisted. It appeared to me that something more, than the mere ossification of the exposed surface of the pulp, took place; and the experiments I have made during the last fourteen months—the result of which I intend to give towards the conclusion of the present article, have revealed to me a most beautiful operation of the economy for the prevention of any bad effects which might otherwise result from the existence of such a vacant space.

From 1834 to 1846, I was in the habit of filling a tooth occasionally, in which the lining membrane was exposed; still, I scarcely thought it possible to preserve the vitality of the pulp by the operation for a great length of time, and hence it was only in cases where the presence of a tooth was called for by some peculiar and urgent necessity, that I attempted it, and then, only under the most favorable circumstances. But in July, 1846, I determined to perform the operation in every favorable case that should occur in my practice, and to keep a record of each, together with the result, or at least, so far as I should be able to ascertain it. This I have done, and will now give a brief statement of the result.

From the fourteenth of August, 1846, to the last of July, 1847, I filled sixty-eight teeth, in which the lining membrane was exposed—forty-seven for females, and twenty-one for males. During the year, I had an opportunity of examining forty-eight of the teeth, at periods varying from two weeks to nine months after the operations had been performed, and in only three of the cases had inflammation and suppuration of the lining membrane and pulp taken place.

In two of these cases, the patients were females, one a married lady, about twenty-eight years of age, and as I have afterwards learned, had been pregnant about three months at the time the operation was performed; the other was a young lady, eighteen years of age, of a chlorotic appearance, with teeth of a soft, chalky texture. The tooth, in the first case, was a right superior central incisor. It caused the patient but little inconvenience, after the third week, for nearly four months, when it became sensitive to the touch, and painful when hot or cold fluids were taken into the mouth. Soon after, it began to assume a dingy, brown color, indicating the loss of vitality. In the second case, the tooth, which was a second molar, on the right side in the upper jaw, continued to be affected with impressions from hot or cold fluids, for two months, when it became very painful, aching severely, and was only relieved by the formation of an alveolar abscess. Soon after, I prevailed on the patient to have the tooth removed.

The subject of the third case, was a gentleman about forty years of age, of a full habit, caused by free living. The tooth, a first left superior bicuspis, continued somewhat painful from the time the operation was performed, until suppuration of the lining membrane and alveolar abscess occurred, which took place the tenth week after it had been filled.

I did not regard the result of the practice, thus far ascertained, as sufficient to enable me to determine definitely its ultimate relative success, although it had exceeded my most sanguine expectations, the time which had elapsed, had not been long enough to enable me to arrive at any positive conclusion upon the subject. I thought it possible that a change in the state of the constitutional health, or the action of some local cause, might so increase the susceptibility of the lining membrane and pulp to morbid impressions, that a diseased action, might, in some way, be excited in them through the conducting medium of the gold filling. Thinking this possible, I embraced every opportunity that occurred, of examining the teeth I had filled after the lining membrane had become exposed. I have seen many of the teeth I filled during the period already referred to, once or twice a year, and sometimes oftener, to the present time. I saw thirty-seven during the years 1849 and 1850, four of which I had not previously had an opportunity of examining since they were filled. Of the sixty-eight filled during the first year, I have subsequently seen sixty, and so far as I have been able to ascertain, loss of vitality has occurred in only five of the teeth. From three of these, I removed the fillings, cleansed the pulp cavities, and filled them very nearly to the extremities of the roots, hoping thereby to arrest the diseased action in their sockets, or at least, so far as to prevent it from extending to the adjacent parts. In one case I was successful, but the teeth in both of the others remained sensitive to the touch; the gums around them continued inflamed and swollen, notwithstanding the most active means were employed to restore them to health. Under these circumstances it became necessary to remove the teeth.

In the five cases in which suppuration of the lining membrane took place, it occurred as nearly as I could ascertain, within twelve months after the operations had been performed.

It is possible, that one or more of the eight teeth not seen since they were operated on, may have lost their vitality, but it is scarcely probable, that if the result was known, it would vary very materially the relative success as shown by the sixty already noticed. It is also possible, that one or more of these may have lost their vitality since I saw them.

From August 19th, 1847, to July 25th, 1848, I filled sixty-two teeth in which the pulps were exposed, forty-six for females and sixteen for males. In three, suppuration of the lining membrane and pulps took place within six weeks from the time the operations were performed. In these cases, the patients were females, one a married lady about thirty years of age; the two others were young ladies—one about sixteen and the other twenty. In the first case, the operation was performed about two months after parturition, while the general system was in a debilitated condition, and more than ordinarily susceptible to morbid impressions. But with this exception, the general health of the patient was good, and her teeth were of a very excellent quality, though rather crowded in their arrangement, and, in consequence of which, the approximal surfaces of several had been attacked by caries. In the left approximal surfaces of the right superior lateral incisor, it had penetrated to the lining membrane. It was removed, however, and the cavity filled without causing much pain, and as it was but slightly affected by cold, strong hopes were entertained that the operation would prove successful. But in about two weeks it became more susceptible to impression from heat and cold, soon after it commenced aching, and in about four weeks from the time it was filled, suppuration of the lining membrane took place.

The constitutional health of the patients in both of the other cases was poor; teeth soft, and the greater part of them more or less affected by caries. In one of these cases the tooth was a superior cuspidatus and had manifested a disposition to ache before it was filled; inflammation soon supervened ,followed by suppuration in about three weeks. In the other case, the tooth was a right second superior cuspidatus, the operation caused but very little unpleasantness, but the tooth was affected with pain when any thing hot or cold was taken into the mouth. At the expiration of six weeks it had assumed a brownish appearance, without, apparently, having caused any diseased action in the socket.

Suppuration and alveolar abscess occurred in a fourth case, about nine months after the tooth was filled. The patient was a married lady, twenty-six years of age; of a sanguino nervous temperament; had suffered severely for upwards of three years from dyspepsia; teeth pearly white, with long and narrow crowns, and of a soft texture. Many of them had decayed and been filled, both before and after her marriage, but the operations had been badly performed, and in nearly every case caries had taken place around the fillings. In the posterior approximal surface of the first left superior bicuspis, it had extended, when she applied to me for professional aid, to the lining membrane; this was considerably wounded in the removal of the decomposed dentine. I succeeded, however, in filling the cavity without causing much pain, but the presence of any thing hot or cold in the mouth occasioned considerable suffering for several days. In a short time, however, the tooth ceased to be affected by these agents, and strong hopes were entertained that the vitality of the tooth would be preserved. But about seven months after, her dyspeptic symptoms having become grately aggravated, she was compelled to abstain altogether from the use of animal diet, and with the increase of the gastric derangement, the susceptibility of the tooth to impressions from heat and cold, returned. It soon became sore and sensitive to the touch, and in about seven or eight weeks it lost its brilliant white appearance; soon after an alveolar abscess formed.

The want of success in the last case resulted, doubtless, from constitutional causes. If the general health of the patient had been good, there is reason to believe that the tooth would not have lost its vitality.

Now, of the sixty-two teeth which I filled over exposed nerves during the second year, I have seen, since the operations were performed, fifty-one, and the four cases already noticed are the only unsuccessful ones that have as yet come to my knowledge. Twenty-three of the fifty-one, I have seen within the last two years; the others I have not seen since the termination of the year in which they were filled. It is possible, therefore, that some of the others may have lost their vitality since I last examined them. The same thing may have occurred to one or more of the eleven teeth which I have not seen since they were filled.

In two of the four unsuccessful cases, I removed the fillings, cleaned and filled the pulp cavities. One of these is still a useful tooth, though there is a discharge of a few drops of purulent matter from the gum, near the extremity of the root, every two or three months. The other tooth was a source of constant suffering, and I extracted it a few weeks after it was refilled.

During the third year, embracing a period from August 23d, 1848, to July 17th, 1849, I filled forty-eight teeth in which the lining membrane was exposed—thirty-three for females, and fifteen for males. In two of these cases, suppuration of the lining membrane and alveolar abscess occurred within two months after the operations were performed. In both, the patients were males—one a gentleman about forty-five years of age; of a full habit, addicted to the use of alcoholic liquors; gums inflamed, spongy, with nearly straight and rounded margins; teeth short and slightly tinged with yellow; of a very good quality, but from neglect several had become affected with caries. In the first left superior molar, the disease had extended from the anterior approximal surface to the pulp cavity. On the removal of the decayed dentine, the lining membrane was exposed and bled so freely, that the cotton used in wiping out the cavity, was almost saturated with blood. But this immediately ceased on the application of a little spirits of camphor to the wounded vessels. The tooth had never ached and the introduction of the filling was effected without causing pain. It was very susceptible, however, immediately after, to impressions from heat and cold, and it soon became sensitive to the touch. In five or six weeks it began to ache; the pain gradually assumed a throbbing character, and at the expiration of two months an alveolar abscess formed. As the tooth continued very sore after the abscess had broke, I advised its removal.

The patient in the other case, was about twenty-three years of age; of a nervous habit, pale complexion, spare, had suffered for several years from dyspepsia; teeth long, well arranged, presenting a pearly-white aspect and rather soft in texture. A number of them had been attacked by caries and several had been filled two years before I saw him, but the operation having been badly performed, the teeth had decayed around the fillings. In the left superior lateral incisor, the disease had penetrated to the pulp cavity. The removal of the decayed dentine from this, and the subsequent operation of filling were attended with considerable pain, but after the operation was performed, the patient experienced but little inconvenience for five weeks. At the expiration of this time, however, it became painful whenever any thing hot or cold was taken into the mouth, but the pain was of so transient a character that it excited but little apprehension on the part of the patient for nearly two weeks, when it became more severe and constant. The tooth, in the mean time, having become sensitive to the touch, I was consulted with regard to it, and directed the application of a leech to the gum. This gave no relief. In a short time the pain became constant, deeper seated, and very soon an alveolar abscess formed.

After the soreness in the tooth had somewhat subsided, I removed the filling, cleansed the pulp cavity, and in about two weeks filled it nearly to the extremity of the root. Since which time, about every two or three months, the tubercle at the extremity of the root suppurates and discharges a few drops of purulent matter through an opening in the gum. But this is the only inconvenience which the patient has experienced from the presence of the tooth.

In one other case, about eleven months after the tooth was filled, suppuration and alveolar abscess occurred. The patient was a lady about forty years of age, of sanguinous temperament, and enjoying very good constitutional health. The crowns of her teeth were long, slightly crowded and perceptibly tinged with yellow near the gums, which latter was regularly festooned and thin along their margins, of a lively rose color, and in every respect, indicative of health. Two or three of her teeth had decayed in their grinding, and several in their approximal, surfaces ; but in most of these places the disease had been completely arrested by gold fillings inserted by a skillful dentist in Philadelphia. But, subsequently to the operation, the posterior approximal surface of the second right superor bicuspis had become the seat of caries, and before she was scarcely aware of the existence of the disease, it had penetrated to the lining membrane. After making a separation with a file between this tooth and the first molar, I removed the decomposed bone and filled the cavity. The operation was attended with some pain, and the tooth was very susceptible to impressions from heat and cold for several days, but as this was not unusual, I did not apprehend any danger. In about two weeks, it entirely ceased to be thus affected, and for nearly nine months it remained free from pain. But, about this time, heat and cold again began to affect it. Soon after it became sore and ached. At the expiration of eleven months the tooth turned dark, and an alveolar abscess formed.

In this case, I was not able to trace the suppuration of the lining membrane and abscess to any constitutional cause, though it may have resulted indirectly from some increase of nervous susceptibility of the general system.

I have seen, up to the present time, thirty-seven of the cases treated during the third year ; and the three already mentioned, are the only ones in which necrosis has resulted from the operation. I would state, however, that I have only seen eighteen of the thirty-seven cases since the termination of the year in which the operations were performed. The result in the eleven cases which I have not seen since the teeth were filled, is, of course, unknown to me.

From August 17th, 1849, to July 22d, 1850, I filled sixty-nine cavities in teeth, in which the lining membrane was exposed ; fifty-four for females, and fifteen for males. In two of the cases, the operation was immediately followed by pain and inflammation of the lining membrane, threatening suppuration. To prevent which, I removed the fillings and applied arsenious acid to the exposed pulps. About three hours after the pain had subsided, the arsenic was removed, together with the pulps, to the extremities of the roots. Two days after, the teeth were filled in the usual manner for operations of this sort. In one case, the tooth has remained free from pain and the socket from manifest disease until the present time ; but, in the other, an alveolar abscess formed about three months after the tooth was filled. As the subsequent discharge of matter has been very small and occurs only every three or four months, it has not been deemed advisable to extract the tooth.

The patient, in one of the above cases, was a lady between twenty-five and thirty years of age, of a sanguino-bilious temperament ; teeth pearly white, rather above the medium size, and not very hard. The tooth operated on, was the first left upper molar. In the other case, the patient was a young gentleman about twenty-one years of age ; the tooth operated on a superior cuspidatus.

Suppuration of the lining membrane and alveolar abscess occurred in two other cases—in one, about four weeks after the tooth was filled ; and in the other, in seven months. The subject of the first case, was a young lady about nineteen years of age, of a strumous habit, with pearly long-crowned teeth, of a soft texture and easily acted on by corrosive agents. She had lost six, and most of the remaining ones had been filled, but the operations not having been properly performed, had not prevented a recurrence of the disease. In the second right inferior molar it had taken place around a large filling in the grinding surface, and in the removal of the softened and decomposed dentine, the lining membrane was exposed to view in two places. As the first molar had been removed, the preservation of this became an object of much importance, and I determined to make an effort to fill it without destoying the pulp.

After having properly prepared, washed and dried the cavity, I placed on the bottom of it two or three drops of a thick solution of gutta percha in chloroform. When the chloroform had evaporated, I proceeded to introduce the filling, which I succeeded in doing without causing any permanent pain. For about two weeks the tooth was exceedingly susceptible to impressions from heat and cold ; but, as it soon ceased to be affected by these agents, I indulged the hope that its vitality would be preserved. In a short time, however, it became sore and slightly raised from its socket. Soon after it began to ache, and from the throbbing sensation experienced, I was convinced that suppuration would soon take place, unless prevented by the immediate removal of the filling and the destruction of the pulp ; but to this operation the patient positively refused to submit, preferring rather to endure the pain than to have the tooth interfered with at that time. At the expiration of a little more than four weeks from the time the tooth was filled, an abscess formed and discharged a large quantity of pus through an opening, which I made with a lancet in the gum, by the side of the tooth, for its escape. I afterwards removed the gold and filled the entire pulp cavity, but the presence of the tooth caused so much irritation, that, ultimately, I advised its removal.

In the other case, the patient was a lady, between thirty-five and forty years of age, of a sanguino-nervous temperament, and for several years had been affected with dyspepsia. Her teeth were of a soft texture, and nearly all were more or less affected with caries. In the right approximal surface of the left superior central incisor, the disease had penetrated to the pulp cavity. I succeeded in filling the tooth without much difficulty, and as it was but very slightly affected with impressions from heat and cold, the operation was regarded as successful. About five months after, the patient had an attack of gastric fever, and during her illness, the tooth become sensitive and slightly painful, which continued after the recovery of her health. At the expiration of seven months from the time it was filled, it assumed a brownish appearance indicating the loss of vitality. Under these circumstances I removed the filling, cleansed the pulp cavity, and in a few days filled the tooth to the extremity of the root. With the exception of a discharge of a few drops of pus, about every two months, from an opening in the gum, it has not since caused the slightest inconvenience, and is but very slightly discolored.

I have seen, up to the present time, forty-seven of the sixty-nine teeth filled during the fourth year, and the four cases just described are the only ones that have come to my knowledge, in which the operation was unsuccessful. In one of these, the suppuration of the lining membrane was, there is every reason to believe, attributable to other causes than the presence of the gold in the tooth. In one other, the operation was performed under such unfavorable circumstances, as almost to preclude even a hope of success.

From September 9th, 1850, to August 13th, 1851, I filled seventy teeth in which the lining membrane was exposed, fifty-eight for females, and twelve for males. Of these, I have seen up to the present time twenty-five, and as yet, so far as I have been able to ascertain, necrosis has occurred in only two of the teeth. The patients in both of these cases were females—one a lady about forty-five years of age, and the other a young lady of eighteen or nineteen.

In the first case, the tooth was a right superior central incisor, and inflammation and suppuration of the lining membrane took place about three weeks after it had been filled. The filling was immediately removed, and in about two days I filled the pulp-cavity and root. Two months have now elapsed, and not the slightest inconvenience has been experienced from the tooth since the last operation was performed. In the other case, the tooth was a left superior cuspidatus, and suppuration of the lining membrane and alveolar abscess occurred about three months after the tooth was filled. This tooth was treated in the same manner as the last, but as another abscess formed soon after the second operation, which discharged more or less every day through a fistulous opening in the gum, causing the patient incessant anoyance, it ultimately become necessary to extract it.

I have now, in as few words as possible, given the result of my observations on filling teeth, when the lining membrane is exposed, together with a few of the details connected with the unsuccessful cases which have occurred in my practice during the last five years. But for convenient reference, I have arranged the cases of each year, with the result, as far as ascertained, in the annexed table.

| | First Year. | Second Year. | Third Year. | Fourth Year. | Fifth Year. | Total. |
|---|---|---|---|---|---|---|
| Number of teeth filled over exposed pulps, | 68 | 62 | 48 | 69 | 70 | 317 |
| For Females, | 47 | 46 | 33 | 54 | 58 | 238 |
| For Males, | 21 | 16 | 15 | 15 | 12 | 79 |
| Number of cases seen after the operation, | 60 | 51 | 37 | 47 | 25 | 220 |
| Successful cases, | 55 | 47 | 34 | 43 | 23 | 202 |
| Unsuccessful cases, | 5 | 4 | 3 | 4 | 2 | 18 |

Now, if all the facts connected with the cases which I have treated, of exposed dental pulp, during the last five years, were known, up to this time, the foregoing report and table, might present a somewhat different result. But if I were, at the same time, to take into account the number of teeth which were filled during this period, under unfavorable circumstances, I do not think they would materially vary it.

When the treatment of teeth in which caries has penetrated to the pulp cavity shall be better understood, it is possible that the vitality of a much larger relative proportion may be preserved. At any rate, so long as it can be done in nine cases out of ten, and even under the most favorable circumstances, I am clearly of the opinion that it should be attempted. A healthy living tooth, is certainly less liable to become offensive to the parts with which it is connected, than one deprived of a very large portion of its vitality. If the lining membrane and pulp did not contribute, in some way, to the preservation of a tooth, and to a *healthy vital connection* between *it* and the *rest of the body*, the process of ossification, as I have stated in another place, would, doubtless, be continued, until the pulp cavity was completely obliterated. But that they may be removed in many cases, without any *immediate* manifest injury, is a fact too well established to admit of doubt. Indeed, by removing the pulp and filling the place which it occupied, a tooth, which otherwise could not be retained without great inconvenience and suffering, may be often rendered serviceable for years, and I believe, in some cases, through life. But as the liability of a tooth to become a morbid irritant is, in accordance with a well established law of the economy, increased, in proportion as its vitality is weakened, the destruction and removal of the lining membrane and pulp should never be determined on, so long as the permanent preservation of the organ can be secured without it.

That the pulp of the tooth, when the operation is successful, undergoes some change, there can be no question. Drs. Harwood of Boston, Foster of New York, and Dwinelle of Cazenovia, are of the opinion, from experiments they have made, that it ossifies. Dr. W. W. Codman, of Boston, says, he has succeeded in many cases, in exciting ossification of the pulp by protecting it from the air with cotton. It is known, too, to nearly all dentists, that the pulps of teeth, under certain circumstances, ossify, and this transition process, is evidently the result of an increase of vascular action in the part, caused by irritation. Teeth in which the crowns have lost a considerable portion of their substance, either from mechanical or spontaneous abrasion, furnish a beautiful example of this process—a wise provision made by nature to prevent the exposure of such a delicate and sensitive tissue. It also sometimes occurs in teeth in which the crowns have suffered no loss of substance. In the latter case, it is doubtless the result of some unknown constitutional or local cause of irritation.

These facts would seem to justify the conclusion that the pulp of a tooth, when subjected, for a sufficient length of time, to the influence of an irritating agent, capable of exciting only a very slight inflammatory action, undergoes ossification; or rather, is converted into a substance resembling *crusta petrosa*, or what Professor Owen terms, *osteo-dentine*. When it fails to undergo this change in a tooth which has been filled after the lining membrane has become exposed, it is liable, from constitutional disease, or other causes, either to perish from derangement of its nutritive functions, or to become the seat of active inflammation, and to suppurate. But something more than mere ossification, or its conversion into *osteo-dentine*, takes place when a space is left between it and the filling. If this vacant space was not obliterated, we have reason to believe that the slightest increase of vascular action would, as has been justly remarked by Dr. Elliot, force a portion of the pulp into it, and thus brought in contact with the sharp angles of the walls of the cavity, active inflammation would be excited, and this, as a natural consequence, would be likely to terminate in suppuration. But nature, ever fruitful in her resources, uses means, as I have satisfactorily ascertained, at least to my mind, by a number of experiments made during the last fourteen months, for the prevention of such an occurrence. They consist, first, in filling the vacant space, with *coagulable lymph*, (*liquor sanguinis,*) effused from the lining membrane or exposed surface of the pulp—then, in its *organization*, and lastly, its *conversion* into *callus* and *bone*, or perhaps more properly,

*osteo-dentine.* Nature seems to employ the same means here, that she does in other parts of the body for the reparation of injuries. But this reproductive process can only take place when the lining membrane or pulp is actually exposed, and from which *plastic lymph* is evidently effused.

That this does actually occur at least in many cases, I am fully convinced, the fact having been most satisfactorily demonstrated to me in three instances, and I have examined other teeth in which there existed the strongest proof that this reparative process had been at work.

In June, 1850, I removed from the posterior approximal surface of the first left superior bicuspis, of Mr. C., a young gentleman about twenty years of age, on several of whose teeth I had operated seven months before, a temporary filling of "Hill's Stopping." At the time I operated on his other teeth, the lining membrane was so badly exposed in this, that I scarcely thought it possible to preserve the vitality of the organ by filling. I determined, however, after having properly prepared the cavity, to attempt to fill it with this compound, intending, if I succeeded, in doing so, to replace it, after it had remained a few months, with gold. After having accurately fitted the end of a small roll of it to the opening of the cavity, I made a deep, conical indentation in that part which, in its introduction, would otherwise have been likely to have come in contact with the exposed part of the pulp. I then forced it gently into the cavity, applying the principal part of the pressure on the outer edges near the walls of the orifice. This done, I pared off with a sharp knife, the protruding parts, and made the surface as smooth as possible. The operation caused a little pain, and the tooth manifested a slight disposition to ache, for two or three hours, but at the expiration of this time, all uneasiness subsided, and in this condition it remained for seven months. On removing the compound, and examining the cavity, which I was able to do very perfectly, as the second bicuspis had been removed previous to the first operation, I discovered to my surprise, a whitish, and apparently semi-translucent substance, slightly elevated from the bottom of the cavity, of a conical shape, with two or three red specks in the centre. I at first, supposed it to be the pulp of the tooth, but on touching it with an instrument discovered that it possessed but very little sensibility, and was almost as hard as cartilage.

Fearing that the contact of gold might be attended with some bad effect, the cavity was refilled with the material used in the first instance, and on removing it at the expiration of six months more, the whitish protuberance at the bottom of the cavity, was found to be solid bone, almost as hard as the surrounding dentinal walls.

In the mean time I removed three gold fillings, two of which had been put in nearly three years before, under like circumstances, and in each case the bottom of the cavity was elevated, presenting the appearance of having been accurately adapted to the inner part of the gold. The other filling had been in only about nine months, as I was informed, and had been put in by a dentist of Bermuda while the patient was a resident of that island. But as the pulp was exposed, a gold cap had been beautifully and accurately fitted to the walls of the inner part of the cavity, which was in the anterior approximal surface of the first right superior molaris, and between which and the lining membrane there had evidently originally existed a considerable vacant space. The filling, however, had not been well condensed; incipient caries had commenced around it, and it was for the removal of this and the prevention of a recurrence of the disease, that it was taken out. In this operation, the gold cap was not disturbed in the slightest degree, and it was with some difficulty that I succeeded in removing it from the cavity. But having done this, I discovered a conical elevation at the bottom of the cavity, corresponding in shape and size to the concavity of the cap. It presented a whitish appearance, with several red specks scattered over the central part of it, and immediately in the center an ossific deposit, with minute spicula of bone radiating in every direction from it, were distinctly seen. On passing a delicate sharp-pointed instrument across this part, it was found to be hard and the grating produced was perceptible both to me and the patient. The other parts were of about the consistence of cartilage or callus, and resembled it in appearance.

After having removed the partially decomposed portions of dentine from the walls of the cavity and replaced the cap, the tooth was refilled. I saw the patient two months after, and up to that time the tooth had not caused the slightest uneasiness.

I have also met with one other case, in which, after the removal of a filling that had been in about ten months, the bottom of the cavity presented a precisely similar appearance. The tooth in this case was a left superior cuspidatus, and the cavity in the right approximal surface.

When this reproductive process does not take place after the operation, it may be owing either to the want of increased vascular action in the lining membrane or pulp, or, to too much inflammation. A certain amount of increased vascular action, seems necessary to the effusion of coagulable lymph, an indispensable requisite to it, but when this is too great, it generally terminates in suppuration. This being the case, it is obvious that the success of the operation must very greatly depend upon the circumstances under which it is performed. However skillful the operator may be in the preparation of the cavity and the introduction of the gold, if these be unfavorable, his efforts to preserve the vitality of the organ, will, in a large majority of cases, prove unavailing. The health of the patient should be unimpaired, the tooth of a tolerably good quality, free from pain at the time the operation is performed; it should never have ached, and the pulp, peridental membranes and surrounding parts should be in a perfectly healthy condition. The cavity, too, should be of a proper shape for the easy introduction and permanent retention of a filling, and the smaller the point of exposure of the lining membrane the greater the prospect of success. It is also important that every particle of completely decomposed dentine should be removed, and if there be any oozing of blood from the ruptured vessels, this should have ceased before the filling is introduced.

But of the cases of exposure of the lining membrane met with by dentists, in teeth possessing sufficient strength of walls to bear the introduction of a substantial filling, probably, not more than one in four, would be favorable for the operation.

The method of procedure which I pursue in filling a tooth when the pulp is exposed, consists, as I have stated in another place; first, in the preparation of the cavity in the usual manner, using the precaution not to wound the lining membrane if it can be avoided, though some of its small vessels are always ruptured in the removal of the innermost layer of dentine, then, to wipe out the cavity with a little raw cotton, moistened with camphorated spirits, which immediately arrests the oozing of blood; next, to introduce the gold, commencing, by placing the folds on one side of the cavity, and inserting fold after fold, without carrying those immediately over the exposed part of the pulp, to the bottom of the cavity, until every part except a small space over the nerve, is thoroughly filled. The folds should be pressed so compactly one against another, as to prevent their inner extremities from being forced against the exposed pulp, in the consolidation of the protruding part of the filling. The gold should now be thoroughly condensed, and the surface finished in a suitable manner.

I have avoided all detail here, as the method is so similar to that which I pursue in filling teeth under other circumstances, and which I have elsewhere described at considerable length.*

I would remark in this connection, that, during the last two or three years, I have occasionally applied a drop of the solution of gutta percha to the bottom of the cavity, and waited until the chloroform had entirely evaporated before I introduced the filling, and in some cases, believe, I have derived advantage from it. At any rate, the teeth seemed less susceptible to impressions from heat and cold. I have also used collodion, but not with as satisfactory results.

Dr. Elliot, of Montreal, a very ingenious and skillful practitioner, states, in an article on "filling teeth over exposed nerves," published in No. 4, vol. 1, new series of the Journal, that he places the gold "directly upon the living nerve, and in

* See 4th edition of Principles and Practice of Dental Surgery.

perfect contact with it, over the whole of its exposed surface," using, when the cavity is sufficiently deep to admit of it, *asbestos*, as a non-conductor, "*enveloped in a few thicknesses of gold foil.*" He moreover says, that within the last year he had "but two cases in which irritation advanced so far as to become troublesome to the patient," and that in "both instances, perfect and permanent relief was obtained by the use of leeches and a mild cathartic."

I had hitherto supposed that the direct contact of any metallic substance with an exposed dental pulp, would, as an inevitable consequence, be followed by irritation and inflammation, but it would seem, from the result of the experiments of Dr. Elliot, that the irritation is principally produced by impressions from heat and cold, conveyed to the nerve of the tooth through the conducting medium of the metal, or from its contact with the sharp angles of the walls of the cavity.

I have not yet tried Dr. E's method, but intend to very soon, and after I shall have given it a fair trial, will report the result.

It has constituted no part of my design, in writing the foregoing article, to treat on any other subject than the *practicability of preserving the vitality of teeth after the lining membrane has become exposed*, but for a very full and comprehensive treatise on "caries of the teeth, complicated with disorders of the pulp and peridental membrane," I will refer the reader to a series of very interesting and ably written papers by Dr. Arthur, now in progress of publication in the Journal. They, no doubt, will embody all that is known upon the subject.

